# Increased Urinary Phthalate Levels in Women with Uterine Leiomyoma: A Case-Control Study

**DOI:** 10.3390/ijerph13121247

**Published:** 2016-12-15

**Authors:** Young Ah Kim, Younglim Kho, Kyoung Chul Chun, Jae Whoan Koh, Jeong Woo Park, Melisa Bunderson-Schelvan, Yoon Hee Cho

**Affiliations:** 1 Department of Obstetrics and Gynecology, Ilsan Paik Hospital, Inje University, 170 Joohwa-ro, Goyang-si, Kyeogi-do 10380, Korea; camanbal@gmail.com (Y.A.K.); kcchun@paik.ac.kr (K.C.C.); J1985@paik.ac.kr (J.W.K.); jwparkmd@paik.ac.kr (J.W.P.); 2 Department of Health, Environment and Safety, School of Human & Environmental Service, Eulji University, 553 Sansung-daero, Seongnam-si, Kyeogi-do 13135, Korea; ylkho@eulji.ac.kr; 3 Department of Biomedical and Pharmaceutical Sciences, College of Health Professions and Biomedical Sciences, University of Montana, 32 Campus Drive, Skaggs 283, Missoula, MT 59812, USA; Melisa.Schelvan@mso.umt.edu

**Keywords:** phthalates, uterine leiomyoma, ∑3-DEHP, ∑4-DEHP

## Abstract

We assessed the urinary concentration of 16 phthalate metabolites in 57 women with and without uterine leiomyoma (*n* = 30 and 27; respectively) to determine the association between phthalate exposure and uterine leiomyoma. To evaluate exposure to di-(2-ethylhexyl) phthalate (DEHP); we calculated the molar sum of DEHP metabolites; ∑3-DEHP (combining mono-(2-ethylhexyl) phthalate (MEHP); mono-(2-ethyl-5-hydroxyhexyl) phthalate (MEHHP); and mono-(2-ethyl-5-oxohexyl) phthalate); ∑4-DEHP (∑3-DEHP plus mono-(2-ethyl-5-carboxypentyl) phthalate); and ∑5-DEHP (∑4-DEHP plus mono (2-(carboxylmethyl)hexyl) phthalate (2cx-MMHP)). The log transformed urinary levels of MEHP; MEHHP; 2cx-MMHP; ∑3-DEHP; ∑4-DEHP; and ∑5-DEHP in the leiomyoma group were significantly higher than those of controls. When we adjusted for age; waist circumference; and parity using multiple logistic regression analyses; we found log ∑3-DEHP (OR = 10.82; 95% CI = 1.25; 93.46) and ∑4-DEHP (OR = 8.78; 95% CI = 1.03; 75.29) were significantly associated with uterine leiomyoma. Our findings suggest an association between phthalate exposure and uterine leiomyoma. However; larger studies are needed to investigate potential interactions between phthalate exposure and uterine leiomyoma.

## 1. Introduction

Myoma, adenomyoma, and endometriosis are estrogen-dependent diseases with unknown etiologies. Uterine leiomyomas are a type of myoma defined as benign tumors of uterine smooth muscles that represent a significant gynecologic disease resulting in reproductive dysfunction and pelvic pain. In particular, uterine leiomyoma is known to be a leading indication for hysterectomy [1,2], and its prevalence is relatively high, although it varies by ethnic group [3,4]. Baird et al. reported that the cumulative incidence of leiomyomas by age 50 in white women is 70% and exceeds 80% in black women in United States [5]. 

Phthalates are man-made chemicals that have been used in various industrial areas and are thought to be estrogenic and androgenic endocrine disruptors, although the evidence is controversial. Human exposure to phthalates is known to occur through inhalation in the workplace, exposure from automobile parts, exposure from building materials, and in the dermal application of personal care products and cosmetics as well as from children’s toys [6,7]. Additionally, exposure occurs through ingestion of water and food contaminated from food processing and packing materials and parenteral exposure through medical devices such as polyvinyl chloride (PVC) tubes and blood bags [8,9]. It has been shown that phthalates are high-volume production chemicals that are rapidly metabolized and excreted from the body [10].

Some phthalates, including di-(2-ethylhexyl) phthalate (DEHP) have been shown to alter or mimic estradiol in vivo and in vitro [11,12,13]. Phthalates prolong the menstrual cycle and increase the rate of premature menopause in animal models [14,15]. However, the adverse effects of phthalates on the human reproductive system are largely unknown. Many studies suggest that phthalates contribute to the pathogenesis of endometriosis [16,17,18]. Similar to endometriosis, leiomyoma are estrogen sensitive; therefore, endocrine-disrupting chemicals may play a role in their development. In fact, phthalates and diethylstilbestrol are reportedly associated with the prevalence of uterine leiomyoma [19,20]. However, there are very few studies examining the effects of phthalates on the development of uterine leiomyoma, and results have been controversial [21,22,23,24,25,26]. In particular, these studies have relied on self-reported fibroids [20,23] or small sample sizes [22,24,25]. The National Health and Nutrition Examination Survey (NHANES, 1999–2004) reported that mono-(2-ethylhexyl) phthalate (MEHP) is inversely associated with uterine leiomyoma, while monobutyl phthalate (MnBP) levels are positively associated with self-reported uterine leiomyoma [20]. Wise and colleagues also reported that the use of hair relaxer, a possible proxy for phthalate exposure, is associated with an increased incidence of self-reported uterine leiomyoma in a cohort of African-American women [23]. However, many women with leiomyomas are not aware of their disease; thus, self-reporting of uterine leiomyoma is a major limitation of these studies [27]. Furthermore, a small case-control study consisting of 36 leiomyoma cases and 29 controls showed that MEHP levels were higher in leiomyoma patients than in controls [24]. Another case-control study with 15 cases and 20 controls found lower serum levels of DEHP in leiomyoma, although the study participants did not present with disease symptoms and were limited to Caucasian women [25]. Recently, an operative cohort study joining 14 clinical centers and consisting of 99 cases and 374 controls reported no significant associations between the phthalates under investigation and odds of a leiomyoma diagnosis in either the unadjusted or adjusted analysis [26]; however, results from previous studies justify further examining the relationship between phthalate exposures and risk of uterine leiomyoma. 

We hypothesized that exposures to DEHP metabolites (including MEHP) and MnBP are associated with risk of uterine leiomyoma. In the present study, we assessed urinary concentrations of 16 phthalate metabolites between women with and without uterine leiomyoma to explore the possible association between phthalate exposure and leiomyoma. 

## 2. Materials and Methods

### 2.1. Clinical Subjects

We recruited patients who had undergone laparoscopic surgery and exploratory laparotomy between March 2013 and July 2015 at the department of Obstetrics & Gynecology in Inje University Ilsan Paik Hospital. Written informed consents were obtained from each patient using consent forms and a protocol approved by the Institutional Review Board of Ilsan Paik Hospital (IB-1211-039). All study participants were initially examined by gynecologic ultrasonography to detect uterine leiomyoma and any other gynecologic problems including intramural, submucosal, and subserosal fibroids before operation. Final uterine leiomyoma diagnoses were confirmed by direct visualization during operation and subsequent pathological analysis. Women with a history of occupational exposure to reproductive toxicants, hormone therapy, any malignancy or other reproductive treatment were excluded from this study. The indication for surgery in the control cases (*n* = 27) was benign ovarian cyst (*n* = 22) and carcinoma in situ (CIS) of the uterine cervix (*n* = 5). 

Initially, the leiomyoma patient group consisted of 49 cases; we excluded cases with pathological evidence of endometriosis (*n* = 9), adenomyosis (*n* = 7), and both endometriosis and adenomyosis (*n* = 3) based on the findings from Huang et al., which showed a significant association between those conditions and an increased level of urinary phthalate metabolites [27]. Therefore, 30 cases of uterine leiomyoma were included in this study.

### 2.2. Demographic Characteristics

The demographic data of cases were obtained from an interviewed questionnaire before operation. The questions included age, body mass index (BMI), waist circumference, age of menarche, duration of menstrual cycle, history of dysmenorrhea and operation, gravity, parity, cigarette smoking, alcohol consumption, exposure to second hand smoke, and exercise.

### 2.3. Urine Collection and Analysis of Urinary Phthalate Metabolites

Gynecologic ultrasonography was used to confirm the presence of fibroids and the need for surgery. Once confirmed, all subjects were requested to undergo blood tests in preparation for surgery and before hospitalization. Urine samples (20–30 mL) were collected in phthalate-free polypropylene containers at the time of blood tests; surgery was scheduled 1–3 days later. Urine samples were immediately centrifuged and stored at −80 °C until further analysis and after measurement of creatinine levels. 

Sixteen urinary phthalate metabolites were analyzed with liquid chromatography electrospray ionization mass spectrometry (LC-MS/MS) using Nanospace SI-2 (Shiseido, Tokyo, Japan) and API 4000 (Applied Biosystems, Foster, CA, USA) as detailed in a previous publication [28]. Standards of phthalate metabolites, some of the compounds listed are not metabolites but original phthalates including MEHP, MnBP, mono-*iso*-butyl phthalate (MiBP), mono-benzyl phthalate (MBzP), mono-cyclohexyl phthalate (MCHP), mono-(2-ethyl-5-hydroxyhexyl) phthalate (MEHHP), mono-(2-ethyl-5-oxohexyl) phthalate (MEOHP), mono-(2-ethyl-5-carboxypentyl) phthalate (5cx-MEPP), mono (2-(carboxylmethyl)hexyl) phthalate (2cx-MMHP), mono-*n*-octyl-phthalate (MnOP), mono-*iso*-nonyl phthalate (MiNP), mono-(3-carboxypropyl) phthalate (MCPP), mono-*iso*-decyl phthalate (MiDP), mono-ethyl phthalate (MEP), mono-cyclohexyl phthalate (MCHP), mono-methyl phthalate (MMP), mono-*n*-pentyl phthalate (MnPP), and their respective ^13^C_4_ or ^13^C_2_-labeled internal standards were purchased from Cambridge Isotope Laboratory (Cambridge Isotope Laboratory, Cambridge, MA, USA). 

The limits of detection (LOD) ranged from 0.01 ng/mL (MCHP) to 2.3 ng/mL (2cx-MMHP). For each instrumental run, reagent blanks and QC samples were included for quality control. The method accuracies were between 80% and 120%, and precisions were lower than 10% relative standard deviation (RSD) for high and low concentrations of spiked samples (*n* = 7).

### 2.4. Statistical Analysis

We summarized the distribution of urinary phthalate metabolites using median and interquartile range. For statistical analysis, Chi-square, Fisher’s exact, and Independent Samples Test were performed using SPSS statistical software (version 14.0, SPSS Inc., Chicago, IL, USA). Phthalate metabolites that were detected at less than 75% were excluded from further analyses. All of the phthalate metabolite data were assessed by Kolmogorov-Smirnov test to evaluate whether they are normally distributed. Since the distribution of phthalate metabolite concentrations was most likely right skewed, all the phthalate metabolite measurements were logarithm transformed to approximate normal distribution. The total concentration of phthalate metabolites was creatinine standardized using the following formula to address urinary dilution as stated in previous studies: Total (phthalate metabolites) = 100 × phthalate (ng/mL)/creatinine (mg/dL) [29,30]. 

DEHP is rapidly metabolized to its monoester, MEHP, which is further extensively modified by various secondary metabolites, including MEHHP, MEOHP, 5cx-MEPP, and 2cx-MMHP. Therefore, to determine total exposure to DEHP, we calculated the urinary level of these metabolites by summing the molar concentrations of MEHP, MEHHP, and MEOHP (∑3-DEHP) as previously described (∑3-DEHP = (MEHP × (1/278.34)) + (MEHHP × (1/294.34)) + (MEOHP × (1/292.33)) nmol/mL) [31,32]. We also calculated the molar sum of 4-DEHP and 5-DEHP by combining the levels of ∑3-DEHP plus 5cx-MEPP (∑4-DEHP = ∑3-DEHP + (5cx-MEPP × (1/308.33)) nmol/mL), and 2cx-MMHP (∑5-DEHP = ∑4-DEHP + (2cx-MMHP × (1/308.33)) nmol/mL), respectively [33,34,35]. In addition, since 5cx-MEPP and 2cx-MMHP are oxidation products with relatively longer elimination half-lives (15–24 h) than those of other DEHP metabolites (approximately 5–10 h) [36], we further calculated the molar sum of 5cx-MEPP and 2cx-MMHP (∑2-DEHP) to determine relatively longer term human exposures to DEHP. 

Multiple logistic regression analyses were performed to estimate odds ratios to determine the association between leiomyoma and the concentration of each urinary phthalate metabolite or the molar sum of DEHP metabolites. The concentration of urinary phthalate metabolites and the molar sum of DEHP metabolites were analyzed with a separate logistic model due to a possible correlation with each other. Covariates for adjustment during the multiple logistic regression were determined based on their significance after univariate analyses (*p* < 0.15); however, BMI was excluded since it is highly correlated with waist circumference. *p* values less than 0.05 were considered statistically significant.

## 3. Results

The demographic characteristics of the controls and cases with uterine leiomyoma are shown in Table 1. There were no differences in dysmenorrhea, smoking, exposure to second hand smoke, and alcohol consumption between the two groups; however, age, waist circumference, and parity were higher in the leiomyoma group than the control group.

Sixteen phthalate metabolites were detected in similar proportion in both the control and leiomyoma group. MnOP, MnPP, MiDP, and MiNP were rarely detected. MCHP was detected at a rate of 18.5% in the control group and 16.7% in the leiomyoma group (Table 2). Therefore, we excluded seven phthalate metabolites (MnBP, MCHP, MCPP, MnOP, MnPP, MiDP, and MiNP) for further analysis because their detection levels were less than 75%.

The log transformed creatinine-adjusted levels of MEHP (1.08 ± 0.14 µg/g creatinine), MEHHP (1.52 ± 0.12 µg/g creatinine), 2cx-MMHP (1.42 ± 0.12 µg/g creatinine), ∑3-DEHP (−0.63 ± 0.12 µg/g creatinine), ∑4-DEHP (−0.46 ± 0.12 µg/g creatinine), and ∑5-DEHP (−0.35 ± 0.12 µg/g creatinine) in the leiomyoma group were significantly higher than those of controls as shown in Table 3 (*p* < 0.05).

When we adjusted for age, waist circumference, and parity using multiple logistic regression analyses, we found log∑3-DEHP (OR = 10.82; 95% CI = 1.25, 93.46) and log∑4-DEHP (OR = 8.78; 95% CI = 1.03, 75.29) were significantly associated with uterine leiomyoma. We also found that logMEHP, logMEHHP and log∑5-DEHP increased the odds of leiomyoma; however, the results were borderline significant (*p* = 0.068, 0.076, and 0.07, respectively) (Table 4). When we further analyzed multiple logistic regression after adjustment for age, waist circumference, parity, and smoking status, log∑3-DEHP (OR = 10.78; 95% CI = 1.24, 93.81) and log∑4-DEHP (OR = 8.61; 95% CI = 1.00, 74.02) were still significant correlated with uterine leiomyoma as shown in Table 4. 

## 4. Discussion

In this study, we measured urinary levels of 16 phthalate metabolites in patients with and without uterine leiomyoma and showed that logMEHP, logMEHHP, log2cx-MMHP, log∑3-DEHP, log∑4-DEHP, and log∑5-DEHP in the leiomyoma group were significantly higher than those in the controls. Log∑3-DEHP and log∑4-DEHP levels were significant factors associated with uterine leiomyoma after multiple logistic regression analysis. LogMEHP, logMEHHP, and log∑5-DEHP also increased the odds of leiomyoma after adjusting for confounding variables; however, the results were borderline significant. 

DEHP is a phthalate that is rapidly metabolized to its monoester, MEHP, and then further modified by various side-chain hydroxylation and oxidation reactions [37,38]. Primary and secondary phthalate metabolites are known to be excreted in urine as glucuronic acid conjugates. In particular, Koch and colleagues reported that 67% of the DEHP dose was excreted in urine after 24 hours and was comprised of MEHHP (23.3%), 5cx-MEPP (18.5%), MEOHP (15.0%), MEHP (5.9%) and 2cx-MMHP (4.2%) [36]. Therefore, urinary phthalate metabolites are suggested to be sensitive biomarkers for evaluating human exposures [39,40,41]. Most toxicity studies have focused on the simple monoester MEHP; however, secondary and oxidized DEHP metabolites can occur at levels 100-fold higher and are suggested to be the ultimate developmental toxicants [42]. Based on metabolism time and half-lives of MEHP, MEHHP, and MEOHP, their presence in the urine reflects short-term exposures, whereas 5cx-MEHP and 2cx-MMHP represent relatively long-term exposures [39]. The relatively long half-lives for elimination make 5cx-MEHP and 2cx-MMHP excellent parameters for measuring the time-weighted body burden of DEHP. In studies examining estrogen-dependent diseases, MEHHP and MEOHP are primarily analyzed [22]; therefore, analysis of 5cx-MEHP and 2cx-MMHP, as well as the molar sum of these metabolites (e.g., ∑4-DEHP, ∑5-DEHP, and ∑2-DEHP) is a relatively new strategy [32,33,34,35]. 

Since phthalates are rapidly metabolized and excreted, phthalate metabolite concentrations detected in a single urine sample represent only recent exposures to the patients. However, many studies have relied upon a single spot urine sample that may not reflect the timing of disease onset and progression. The nearly ubiquitous presence and high volume production of short-lived chemicals, such as the phthalates, suggests continual human exposure despite their short-lived nature [41,43]. Creatinine-corrected urinary concentrations of MEP have been moderately reproducible (intraclass correlation coefficient (ICC) > 0.48) across 2–4 week sampling intervals among studies in women of reproductive age [44,45]. In addition, creatinine-corrected urinary concentrations of MBzP have high temporal reliability (ICC > 0.53) across studies of various populations evaluating time intervals ranging from 8 days to 6 months [44,45,46,47,48]. Despite a lack of temporal reliability for urinary DEHP metabolites (ICC = 0.13–0.22) compared to other metabolites with longer half-lives, the data reported here suggests that a single sample may be representative of exposure levels over time, particularly if exposure is consistent [41].

Five previous studies evaluated phthalate metabolites and uterine leiomyoma. In a cross-sectional study utilizing NHANES data, Weuve et al. reported that MEHP is inversely associated with uterine leiomyoma, while MnBP levels are positively associated with self-reported uterine leiomyoma [20]. Wise et al. relied on proxy exposure assessment and self-reported outcomes [23], and Luisi et al. evaluated phthalate levels in the blood [25]; however, methodological issues were identified in both studies. Phthalates are quickly hydrolyzed to their monoesters leading to higher phthalate monoesters; therefore, phthalates in blood may not accurately reflect internal dose. As such, concentration of phthalates in urine is the best matrix to assess the level of phthalate exposure [49]. Although a small case-control study consisting of 36 leiomyoma cases and 29 controls, Huang et al. found that urinary ∑DEHP is associated with an increased risk of leiomyoma after adjusting for the *GSTM1* genotype, which is the first study to explore the gene-environmental interaction of phthalate exposure and *GSTM1* polymorphisms [24]. Several studies using urine samples failed to show a constant association between urinary phthalate concentrations and uterine leiomyoma [25,26]. Recently, in an operative cohort study joining 14 clinical centers and consisting of 99 cases and 374 controls, Pollack et al. did not find an association between phthalates and uterine leiomyomas [26]. However, our results show a positive association between urinary ∑DEHP and uterine leiomyoma despite a smaller sample size. These discrepancies may be explained by differences in patient recruitment methodologies; Pollack et al. recruited patients diagnosed during laparoscopy or laparotomy only. Direct surgical visualization can miss intramural, submucosal, and subserosal fibroids, the most common fibroid types. In contrast, we recruited uterine leiomyoma patients without any other medical issues, including endometriosis and/or adenomyosis, after confirming the diagnosis with gynecologic ultrasonography as well as direct visualization during operation and subsequent pathological analysis. Cases with uterine leiomyoma only had significantly higher levels of urinary DEHP metabolites (log∑3-DEHP and log∑4-DEHP) than controls, suggesting that exposure to phthalates might affect the pathogenesis of leiomyoma.

Numerous animal studies have reported that phthalates induce dysfunction of the reproductive system, mainly in male animals, by inhibiting androgen production and steroidogenesis pathways [50,51,52]. However, several in vitro and in vivo studies showed estrogenic effect of phthalates [14,53,54], although the estrogenic activities of phthalates are still controversial and may differ by species, sex, time, and dose [55,56,57,58]. Previous studies have reported estrogenic effects of DEHP in fish [53,59] and mammals [14,60,61], and these estrogenic effects are suggested to be attributable to stimulation of aromatase (*CYP19*) and an increase in *Vtg* expression, which increases estrogen levels. Furthermore, Borch et al. reported an increase in anogenital distance (AGD) in female rats [55] after in utero DiBP exposure. Another study by Borch et al. showed decreased PPARγ protein levels in prenatally DEHP-exposed female rats [62]. A study by Boberg et al. also showed similar results; DiBP increased AGD and ovarian aromatase mRNA levels and decreased hepatic gene expression of *PPARα* in female rats [56]. PPARs as a super family are known to be involved in various physiological pathways including reproductive hormone synthesis, fatty acid metabolism, insulin function, immunology, central nervous system functions, and cancer development [63,64]. In particular, expression of *PPARγ* in granulosa cells is involved in various ovarian functions [65], and activation of the aromatase enzyme, which is responsible for the final conversion of testosterone to 17β-estradiol [66], is mediated by PPARγ [67,68]. Therefore, phthalate exposure may interfere with *PPARγ* expression, resulting in elevated aromatase and estradiol levels. 

Moreover, aromatase is stimulated by follicle-stimulating hormone (FSH) in rat, ruminants, and humans [69,70], acting mainly through the cAMP/protein kinase A [71]. An animal study by Gonzalez-Robayna et al. showed that FSH also activates protein kinase B through phosphatidylinositol 3-kinase (PI3K) in rat granulosa cells [72], and granulosa cell differentiation and aromatase expression require activation of this pathway [73,74]. Therefore, intracellular signaling by gonadotrophic hormones for stimulation of aromatase expression/activity and estradiol accumulation is a complex process involving many potential pathway interactions. Recently, a murine study by Hannon et al. showed that DEHP disrupts estrous cyclicity and accelerates primordial follicle recruitment through dysregulation of PI3K signaling [54]. DEHP was shown to selectively increase mRNA levels of the stimulatory PI3K signaling factors, including *Mtorc1* and *Pdpk1* [54]. Therefore, according to their findings, DEHP promotes the PI3K signaling pathway to accelerate aromatase activity for more estrogenic activities. In particular, the PI3K signaling pathway is involved in cell proliferation, survival, migration, metabolism, and ovarian folliculogenesis [75,76,77]. Furthermore, Sharma and Singh reported that PPARγ is involved in FSH and PI3K signaling pathways for developing polycystic ovarian syndrome in response to rosiglitazone, an insulin sensitizer [78]. Taken together, metabolites of DEHP may be associated with estrogen-dependent diseases such as uterine leiomyoma that result from an increase in aromatase and estradiol levels attributable to dysregulation of PI3K signaling and PPARγ activity. 

In our study, we found that age and waist circumference are potential risk factors for uterine leiomyoma. The age finding is consistent with other studies [21]; however, in this study, alcohol, and dysmenorrhea were not determined to be risk factors for developing uterine leiomyoma. This may be attributed to the small sample size, which limits our interpretation and justifies conducting larger studies. LogMEHP, logMEHHP and log∑5-DEHP increased the odds of leiomyoma after multiple logistic regression analysis with borderline significance, which is likely attributable to the small sample size. Furthermore, levels of urinary DEHP metabolites (log∑3-DEHP and log∑4-DEHP) showed a significantly higher odds ratio of uterine leiomyoma after adjustment for other risk factors, including age, waist circumference, parity, and smoking; however, the small sample sized used in this study resulted in very wide confidence intervals. Consequently, the small sample size should be considered when interpreting these results and merits caution. In addition to small sample size, there are other limitations of this study. For example, we analyzed phthalate metabolites in single, spot urine samples collected after the onset of symptoms in cases with leiomyoma. The natural history of uterine leiomyoma is complex and can have notable growth in the six months prior to detection [79]. Therefore, phthalate levels measured at the time of diagnosis may reflect a relevant period at least for their short-term growth. The comparison of phthalates between cases and controls who all have medical reasons warranting surgery that could be associated with other phthalate exposures is another limitation of this study. For example, parenteral exposure to phthalates may occur through medical devices such as PVC tubes and blood bags [9]. However, we collected urine samples under the same conditions in both case and control cases prior to each operation. Therefore, the effect of parenteral exposure to phthalates would be negligible. In addition, the frequency of phthalate exposure in controls would vary depending on the selection of controls and the indications for surgery. We divided case and control groups according to the existence of myoma, and we further excluded cases with pathological evidence of adenomyosis or endometriosis. We also excluded women with a history of occupational exposure to reproductive toxicants, hormone therapy, and any malignancy or other reproductive treatment in both case and control groups to minimize this effect. Despite these precautions, our controls were diagnosed with gynecologic conditions such as benign ovarian cyst and carcinoma in situ of the uterine cervix. Although we found no evidence in the literature that phthalate exposure is associated with these diseases [80,81], we cannot exclude the possibility that the medical necessity for surgery in control cases was associated with phthalate exposure. In addition, we did not have age-matched controls for the cases with leiomyoma. Leiomyomas are generally detected upon the onset of symptoms among women in the 4th and 5th decade of life. Therefore, we used age as a covariance in the regression model. Finally, we used a hospital-based control group instead of a population-based control group. However, other studies have also relied on a patient control group without uterine leiomyoma. 

## 5. Conclusions

Despite these limitations, we found a significant increase in the risk of uterine leiomyoma in subjects with higher levels of total urinary DEHP metabolites (log∑3-DEHP and log∑4-DEHP) and increased age. Our findings suggest that there is an association between phthalate exposure and uterine leiomyoma. However, larger studies are warranted.

## Figures and Tables

**Table 1:** Clinical characteristics of cases with uterine leiomyoma and controls.

Variable	Control (*n* = 27)	Cases (*n* = 30)	*p*-Value
Age (years)	34.78 ± 1.90	42.67 ± 1.02	<0.001
BMI (kg/m^2^)	22.06 ± 0.68	23.06 ± 0.54	0.08
Waist circumference (cm)	70.06 ± 1.06	74.44 ± 1.30	0.01
Gravida			
0	8 (29.63)	5 (16.67)	0.49
1–2	10 (37.04)	12 (40.0)	
3 and more	9 (33.33)	13 (43.33)	
Parity			
0	12 (44.44)	7 (23.33)	0.02
1	7 (25.93)	3 (10)	
2 and more	8 (29.63)	20 (66.67)	
Dysmenorrhea *	9 (33.33)	12 (40)	0.60
Cigarette smoking *	9 (33.33)	5 (16.67)	0.14
Second-hand smoking *	10 (37.04)	9 (30)	0.36
Alcohol consumption *	26 (96.29)	29 (96.67)	0.94

The data are presented as the mean ± SE or numbers (%); *p* values were calculated by *t*-test or *x*^2^-test. Fisher’s exact test was used when individual cells were <5; * indicates “Yes”.

**Table 2:** Laboratory measurement of urinary phthalate metabolite concentrations and distribution by case status.

Phthalate Metabolite	Control		Cases	
	Measured > LOQ *n* (%)	Median (IQR) (µg/g Creatinine)	Measured > LOQ *n* (%)	Median (IQR) (µg/g Creatinine)
MEHP	26 (96.29)	4 (2.68–7.66)	28 (93.33)	5 (2.54–29.25)
MEOHP	27 (100)	6.16 (4.48–10.09)	30 (100)	8.55 (6.14–18.35)
MEHHP	27 (100)	16.74 (11.63–25.36)	30 (100)	17.62 (13.76–37.81)
2cx-MMHP	27 (100)	12.89 (10.42–19.87)	30 (100)	17.59 (11.34–34.06)
5cx-MEPP	27 (100)	16.21 (12.74–22.84)	30 (100)	17.94 (12.24–34.40)
MnBP	13 (48.14)	0 (0–12.63)	21 (70.00)	11.62 (0–19.23)
MBzP	25 (92.59)	4.7 (2.16–7.83)	26 (86.67)	5.07 (1.55–10.08)
MiBP	27 (100)	4.11 (2.37–7.50)	30 (100)	3.93 (2.61–6.14)
MEP	24 (88.89)	4.87 (2.42–12.10)	25 (83.33)	3.96 (1.38–14.60)
MMP	24 (88.89)	4.18 (1.55–6.64)	26 (86.67)	3.98 (1.06–16.69)
MCHP	5 (18.52)	NA	5 (16.67)	NA
MnOP	0	NA	1 (3.33)	NA
MCPP	19 (70.37)	1.61 (0–4.01)	16 (53.33)	1.46 (0–6.31)
MnPP	0	NA	1 (3.33)	NA
MiDP	0	NA	0	NA
MiNP	0	NA	0	NA

LOQ = limit of quantitation; IQR = interquartile range; NA = Not available.

**Table 3:** The log transformed creatinine-adjusted urinary phthalate levels in cases and controls.

Urine Level (µg/g Creatinine)	Control (*n* = 27)	Cases (*n* = 30)	*p*-Value
MEHP	0.68 ± 0.06	1.08 ± 0.14	0.02
MEOHP	0.91 ± 0.05	1.17 ± 0.13	0.08
MEHHP	1.23 ± 0.05	1.52 ± 0.12	0.04
2cx-MMHP	1.16 ± 0.04	1.42 ± 0.12	0.04
5cx-MEPP	1.24 ± 0.04	1.47 ± 0.12	0.11
∑3-DEHP *	−0.98 ± 0.05	−0.63 ± 0.12	0.01
∑4-DEHP **	−0.79 ± 0.04	−0.46 ± 0.12	0.02
∑5-DEHP ***	−0.67 ± 0.04	−0.35 ± 0.12	0.02
∑2-DEHP ****	−0.97 ± 0.03	−0.73 ± 0.12	0.07
MBzP	0.68 ± 0.07	0.69 ± 0.08	0.95
MiBP	0.60 ± 0.07	0.64 ± 0.06	0.66
MEP	0.83 ± 0.09	0.85 ± 0.13	0.87
MMP	0.60 ± 0.09	0.74 ± 0.14	0.40

All phthalate metabolites were creatinine (mg/dL) standardized. The data are presented as the mean ± SE; * ∑3-DEHP is the molar sum of MEHP, MEHHP, and MEOHP; ** ∑4-DEHP is the molar sum of MEHP, MEHHP, MEOHP, and 5cx-MEPP; *** ∑5-DEHP is the molar sum of MEHP, MEHHP, MEOHP, 5cx-MEPP, and 2cx-MMHP; **** ∑2-DEHP is the molar sum of 5cx-MEPP and 2cx-MMHP.

**Table 4:** Odds ratio and 95% confidence interval (CI) for the association between leiomyoma and log transformed creatinine-adjusted urinary phthalate levels.

Urine Level (µg/g Creatinine)	Crude OR (95% CI)	Adjusted OR ^a^ (95% CI)	Adjusted OR ^b^ (95% CI)
MEHP	3.75 (1.17–12.0)	4.08 (0.89–18.60)	4.17 (0.90–19.40)
MEOHP	2.83 (0.81–9.87)	3.82 (0.71–20.60)	3.71 (0.71–20.80)
MEHHP	3.63 (0.93–14.20)	5.43 (0.84–35.20)	5.52 (0.83–36.57)
2cx-MMHP	3.87 (0.86–17.30)	3.08 (0.63–15.10)	3.10 (0.63–15.12)
5cx-MEPP	2.76 (0.73–10.37)	2.95 (0.58–15.02)	2.95 (0.58–14.96)
∑3-DEHP *	6.04 (1.24–29.37)	10.82 (1.25–93.46)	10.78 (1.24–93.81)
∑4-DEHP **	5.73 (1.10–29.74)	8.78 (1.03–72.29)	8.61 (1.00–74.02)
∑5-DEHP ***	6.18 (1.07–35.86)	8.24 (0.86–79.10)	8.01 (0.84–76.0)
∑2-DEHP ****	3.53 (0.78–15.84)	3.29 (0.58–18.63)	3.30 (0.59–18.55)
MBzP	1.06 (0.23–5.80)	1.27 (0.16–6.81)	1.17 (0.17–8.18)
MiBP	1.42 (0.31–6.54)	2.12 (0.29–15.42)	2.15 (0.29–15.80)
MEP	1.09 (0.39–3.07)	1.27 (0.36–4.44)	1.63 (0.39–6.75)
MMP	1.51 (0.58–3.95)	1.26 (0.40–3.95)	1.15 (0.35–3.84)

All phthalate metabolites were creatinine (mg/dL) standardized. **^a^** Adjusted for age, waist circumference, and parity; **^b^** Adjusted for age, waist circumference, parity, and smoking status; * ∑3-DEHP is the molar sum of MEHP, MEHHP, and MEOHP; ** ∑4-DEHP is the molar sum of MEHP, MEHHP, MEOHP, and 5cx-MEPP; *** ∑5-DEHP is the molar sum of MEHP, MEHHP, MEOHP, 5cx-MEPP, and 2cx-MMHP; **** ∑2-DEHP is the molar sum of 5cx-MEPP and 2cx-MMHP.
